# Food Processing Technologies to Develop Functional Foods With Enriched Bioactive Phenolic Compounds in Cereals

**DOI:** 10.3389/fpls.2021.771276

**Published:** 2021-11-30

**Authors:** Deepak Kasote, Rhowell N. Tiozon, Kristel June D. Sartagoda, Hameeda Itagi, Priyabrata Roy, Ajay Kohli, Ahmed Regina, Nese Sreenivasulu

**Affiliations:** ^1^Centre of Excellence in Rice Value Addition (CERVA), International Rice Research Institute (IRRI)—South Asia Regional Centre (ISARC), Varanasi, India; ^2^International Rice Research Institute, Los Baños, Philippines; ^3^Max-Planck-Institute of Molecular Plant Physiology, Potsdam-Golm, Germany

**Keywords:** cereals, phenolics, flavonoid, anthocyanin, pigmented cereals, post-harvest process

## Abstract

Cereal grains and products provide calories globally. The health benefits of cereals attributed to their diverse phenolic constituents have not been systematically explored. Post-harvest processing, such as drying, storing, and milling cereals, can alter the phenolic concentration and influence the antioxidant activity. Furthermore, cooking has been shown to degrade thermo-labile compounds. This review covers several methods for retaining and enhancing the phenolic content of cereals to develop functional foods. These include using bioprocesses such as germination, enzymatic, and fermentation treatments designed to enhance the phenolics in cereals. In addition, physical processes like extrusion, nixtamalization, and parboiling are discussed to improve the bioavailability of phenolics. Recent technologies utilizing ultrasound, micro- or nano-capsule polymers, and infrared utilizing processes are also evaluated for their effectiveness in improving the phenolics content and bio-accessibility. We also present contemporary products made from pigmented cereals that contain phenolics.

## Introduction

Genetic diversity present in cereals is an excellent source of a variety of bioactive chemicals. The polyphenolic molecules in cereal grains include phenolic acids (ferulic acid, ferulic, vanillic, p-coumaric, caffeic, protocatechuic, p-hydroxybenzoic, genistic, chlorogenic, and syringic acids), flavonoids (flavones, flavonols, isoflavones, flavanols, flavanones, anthocyanins), and avenanthramides. Given that some of these compounds are water-soluble and others are insoluble antioxidants, strategies for increasing their bioavailability must be investigated (Jones et al., 2019). Cinnamic acid and its derivatives, especially ferulic acid, are the main phenolic acids found in cereal grains (Zhou et al., 2005). Quercetin glycosides, flavones, and flavonols are the major flavonoids found in cereal grains; however, anthocyanins contribute substantially to the overall flavonoid content of dark-colored grains such as purple maize and purple rice (Francavilla and Joye, 2020). Buckwheat grains have also been shown to contain catechins. Sorghum is one of the few kinds of cereal that contains large amounts of oligomeric and polymeric flavonoids known as condensed tannins (Espitia-Hernández et al., 2020). Purple and red pigmentation in cereals is linked to larger amounts of polyphenols covering a wide array of bioactive compounds such as anthocyanins, proanthocyanidins, flavonoids, phenolic acids, and lignins (Lachman et al., 2018; Mbanjo et al., 2020; Loskutov and Khlestkina, 2021).

The majority of phenolic acids exist in conjugated and bound forms, mostly in the bran (Kim et al., 2006; Chandrasekara and Shahidi, 2010). Lignans, a class of polyphenols with significant health benefits, especially as precursors to mammalian lignans, are plentiful in cereal grains. Whole grain cereals and their products are well-known for containing more protein, minerals, vitamins, dietary fiber, and phytochemicals than their polished counterparts. In cereals, a large proportion of phenolic compounds are concentrated in the bran, where they are typically found as soluble conjugates or covalently linked to sugar moieties or cell wall structural components (Mnich et al., 2020). By cross-linking to lignin, extensins, and glucuronoarabinoxylan components, bound phenolic acids are usually implicated in the structure of the cell wall. In white, red, and black rice grains, ferulic, p-coumaric, syringic, and isoferulic acids are found in bound forms (Shao et al., 2014). Further, they may be physically trapped inside cereal matrices and intact cells (Acosta-Estrada et al., 2014; Wang et al., 2014a; Wang Z. et al., 2020). Due to the fact that these chemicals are often found bonded, organic solvents cannot usually remove them. When coupled with increased dietary fiber content in whole-grain foods, increased levels of bioactive phytochemicals such as phenolic compounds, sterols, tocols, and lignans have been found to protect against non-communicable illnesses (Derrien and Veiga, 2017). Given that the bulk of phenolics are contained in the bran or seed coat of grains, it is more beneficial to consume whole grains and intact seed-based meals. Numerous *in vivo* and *in vitro* studies on the health benefits of cereals have clearly shown that diets high in whole grain cereals and cereal-based products contribute to the prevention of a range of chronic diseases with significant public health implications (Chen et al., 2019; Francavilla and Joye, 2020).

Numerous variables may affect the content and possible health benefits of meals along the value chain. The concentration of these bioactives in cereals, food components, and dietary supplements, all of which can have an effect on human health and wellbeing, may be influenced by genetics, growing and storage conditions, post-harvest treatments, food formulation, and processing. Postharvest processing of cereal grains is critical to making them preferentially edible and biofunctional and impart distinctive features for better cooking and organoleptic aspects (Oghbaei and Prakash, 2016). These processes include drying, grinding, and storing, contributing to the product’s shelf life, stability, and palatability. Numerous cooking techniques, particularly thermal processing techniques such as microwave heating, roasting, frying, steaming, autoclaving, boiling, extrusion, and baking, have been extensively used to improve the palatability and taste of cereal grains and their products. Several pre-and post-harvest methods have been devised to liberate bound phenolics from the indigestible matrix and boost the free-soluble phenolics content (Wang et al., 2014b; Călinoiu and Vodnar, 2019). The effects of various pretreatment procedures on specific phenolic compounds are shown in Table 1, while Supplementary Table 1 summarizes the change in the antioxidant capacity of the total phenolic content. Understanding the effect of various processing methods on phenolic compounds in grain is critical for maintaining or enhancing these compounds’ health-promoting qualities in processed cereal products (Kadiri, 2017).

**TABLE 1:** Individual phenolic compounds as affected by various pre-treatment methods.

**Author**	**Botanical source**	**Processing/postharvest method/s applied**	**Ferulic acid**	**Syringic acid**	**Caffeic acid**	**Protocatechuic acid**	**Chlorogenic acid**	**Hydroxybenzoic Acid**	**Vanillic acid**	**Coumaric acid**	**Rutin**	**Sinapic acid**	**Quercetin**
Chen et al. (2019)	Rice	Extrusion	↑F↑B↑T	↑F↑B↑T	↑F↑B↑T	↑F↑T	↑F↑B↑T	↑F↑B↑T		↑F↑B↑T			
Zeng et al. (2016)	Rice		↑F↓B	↑F↓B	↓F↑B		↓F↑B	↓F↑ B	↓F↑B	↓F↓B		↑F ↑B	
	Wheat		↑F↑B	↑F↑B	↓F↓B		↓F↑B	↓F↑B	↓F↑B	↑F↓B		↑F↑B	
	Oat		↑F↑B	↑F↑B	↓F↑B		↓F↑B	↓F↑B	↑F↓B	NCF↓B		↑F↑B	
Ti et al. (2014)	Rice	Germination	↑T	↑T	↑T	↑T	↓T						
Kim et al. (2018)	Wheat		↑T	↑T	↑T			↑T	↑T	↑T			
Xiang et al. (2017)	Corn		↑F↑B↑T	↑F↑B↑T			↑T ↑F ND-B						
			↑F↑B↑T	↑F↑B↑T			↑T ↑F ND-B						
Ge et al. (2021)	Barley	Germination	↑T		↑T	↑T	↑T	↑T			↑T		↑T
		Infrared drying	↑T		↑T	↑T	↑T	↑T			↑T		↑T
Liu et al. (2017)	Rice	Enzymatic treatment	↑T↑F↑SC	↓T↑F↓SC	↑T↑F↑SC	↑T↑F ↑SC	↑T↑SC ↑ND-F	↑T↑F↑SC		↑T↑F↑SC			↑T↑F ↑SC
Cho et al. (2018)	Corn	Enzymatic treatment	↑T	↑T	↑T			↑T	↑T	↑T		↑T	
		Enzymatic treatment	↑T	↑T	↑T			↑T	↑T	↑T		↓T	
Bei et al. (2018)	Oat	Fermentation	↑F↑B		↓F↑B		↑F↑B	↓F↑B B	↑F ND-B	↑B ND-F	ND-F ND-B	↓F↑B	ND-F ↓B
		Fermentation followed by enzymatic treatment	↑F↑B		↑F↑B		↑F↑B	↑F↑B	↑F ND-B	ND-F ↑B	ND-F ND-B	↑F↑B	ND-F ↑B
Amaya Villalva et al. (2018)	Wheat	Fermentation and enzymatic treatment	↓T↑F↓B										
		Fermentation and enzymatic treatment followed by baking	↓T↑F↓B										
Skrajda-Brdak et al. (2019)	Wheat	Fermentation	↑F	↑F				↑F	↑F	↑F		↑F	
	Rye		↑F	ND-F				↑F	↑F	↑F		↑F	
	Spelt		↑F	↓F				↑F	↑F	↑F		↑F	
Chen et al. (2019)	Rice	Fermentation	↑F↑B↑T	↑F↑B↑T	↑F↑B↑T	↑F↑T ND-B	↑F↑B↑T	↑F↑B↑T		↑F↑B↑T			
Călinoiu et al. (2019)	Wheat	Fermentation followed by ultrasound	↑T					↑T	↑T			↑T	
	Oat		↑T		↑T	↑T		↑T	↑T	↑T		↑T	
Gabaza et al. (2016)	Millet	Fermentation	↑F↓B		ND-F ↓B	↑F↓B				↑F↓B		↑F↑B	
N’Dri et al. (2013)	Sorghum	Hydrothermal treatment	↑F↓B	ND-F ↑B	↑F↓B	↑F↓B	↑F↓B		↑F↑B	↑F↑B		ND-F ↑B	
	Fonio		↓F↑B	ND-F ↑B	↓F↑B	↓F↑B	↓F↑B		↓F↑B	↓F↑B		↓F↑B	
	Millet		↑F↑B	ND-F ↑B	↑F↓B	↓F↓B	↑F↓B		↓F↑B	↑F↑B		ND-F ↓B	
Chmiel et al. (2018)	Rice	Microwave	↑T	↑T	↑T	↑T	↑T	↑T	↑T	↑T		↑T	
Wanyo et al. (2014)	Rice	Infrared	↑T	↑T	↑T	↑T	↑T	ND-T	↑T	↑T	ND-T	↑T	ND-T
		Enzymatic treatments	↓T	↑T	↑T	↑T	↑T	ND-T	↑T	↑T	ND-T	↑T	ND-T
Xu et al. (2015)	Rice	Extrusion	↓T↓F↓B	↓T↓F↓B	↓T↓F↓B		↓T↓F ND-B	↓T↓F↓B	↓T↓F↓B	↓T↓F↓B		↓T↓F ND-B	
		Enzymatic treatment	↓T↑F↓B	↓T↓F↑B	↑T↓F↓B		↓T↓F ND-B	↑T↑F↓B	↓T↓F↓B	↑T↑F↑B		↓T↓F ND-B	
Călinoiu et al. (2019)	Wheat	Thermal processing followed by ultrasound	↑T		↑T			↑T	↑T	↑T		↑T	
	Oat		↑T		↑T			↑T	↑T	↑T		↑T	
Chen et al. (2018)	Oat	Ultrasound	↑F↑ SC↓B		↓F↑SC↓B	↑F↑SC		↑F	↓F	↑F↓B			
Setyaningsih et al. (2019)	Red Rice	Ultrasound	↓T	↑T	NC-T	↑T	↑T		↑T	↑T			NC-T
	Black Rice		↓T	↓T	ND-T	↑T	ND-T		↑T	↓T			↑T
Hassan et al. (2020)	Sorghum	Ultrasound	↑T(T1), (T2) ↓T(T3), (T4)										↑T(T1),(T2) ↓T(T3),(T4)
Lohani and Muthukumarappan (2016)	Sorghum	PEF	↑T		↑T	↑T		↑T		↑T			
Belén Martín-Diana et al. (2021)	Wheat	Microencapsulation	↑F										
			↑F										
Martini et al. (2017)	Wheat	Micronization	↑T					↑T	↑T	↑T		↑T	
Wang et al. (2014b)	Corn	Microfluidization	↑T							↑T			
Gaytán-Martínez et al. (2017)	Red Sorghum	Nixtamalization	↓T				↓T						
	White sorghum		ND-T				↓T						
Buitimea-Cantúa et al. (2018)	Corn sorghum	Nixtamalization and extrusion	↓B (ENCF) ↓B (Tortillas ENCF) ↑B (ENCF with sorghum added after extrusion) ↓B (Tortillas ENCF with sorghum added after extrusion)							↓B (ENCF) ↓B (Tortillas ENCF) ↑B (ENCF with sorghum added after extrusion) ↑B (Tortillas ENCF with sorghum added after extrusion)			

*ABTS, 2,2′-azino-bis (3-ethylbenzothiazoline-6-sulfonic acid); B, Bound; BCR, Black Colored Rice; DPPH, 2,2-diphenyl-1-picryl-hydrazyl-hydrate; ENCF, Extruded Nixtamalized Corn Flour; F, Free; FRAP, Ferric reducing antioxidant power; ND, Not Detected; PEF, Pulsed electric Field; RCR, Red Colored Rice; SC, Soluble Conjugate; T, Total; TAC, Total Anthocyanin Content; TFC, Total Flavonoid Content; TPC, Total Phenolic Content.*

**FIGURE 1:**
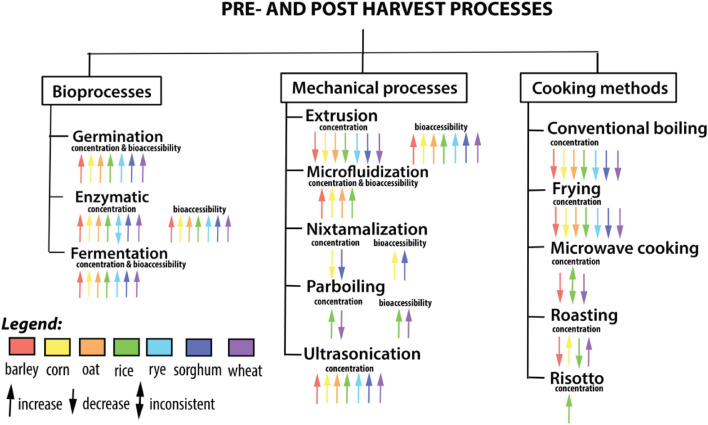
Influence of bioprocesses, mechanical processes, and cooking methods on the concentration and bioavailability of phenolic compounds.

Most of the processing approaches alter the polyphenol composition of cereals and their products and improve the availability and digestibility of phenolics due to the chemical or physical modifications that occur during processing (Oghbaei and Prakash, 2016; Ribas-Agustí et al., 2017). In comparison, several of these post-harvest processing methods have been shown to degrade the natural phenolic components in the end products, resulting in decreased bioavailability during *in vivo* digestion (Ribas-Agustí et al., 2017). Hence in this review article, we detail the chemical diversity of bioactives identified in cereal crops and comprehensively review the implications of (a) bioprocessing methods such as germination, enzymatic treatment, and fermentation, and (b) novel technological processing methods such as ultrasonication, parboiling, micro- and nano-encapsulation, infrared, and pulse electric field methods for improving the stability of phenolic compounds in functional foods and for specific delivery of cereal phenolic compounds with increased bioavailability (Figure 1). To collect as many relevant citations as feasible, a broad variety of scientific databases were searched. Only data from literature published until 31st August 2021 obtained from the following databases (Google Scholar, PubMed, SciELO, and Scopus) were included in this review. To this end, the following keywords and their combinations were used: cereal, grains, wheat, rice, corn, barley, oat, rye, millet, sorghum, phenolics, phenolic acids, flavonoids, anthocyanin, processing, post-harvest, thermal, extrusion, nixtmalization, microwave, ultrasound, microencapsulation, micronization, microfluidization, enzyme, fermentation, baking, pulsed electric field, product, development, cardiovascular disease, obesity, inflammation, diabetes, or glucose.

## Phenolic Compound-Rich Bioactive Ingredients in Cereals for Sustained Human Health

Recent years have seen the discovery of novel functions for phenolic chemicals, especially flavonoids. Indigenous farmers have preserved the medicinal characteristics of grain types, landraces, cultivars, and wild forebearers for their therapeutic potential. These traits are now attracting considerable scientific attention. For example, Njavara, a Kerala medicinal rice, is widely utilized in Ayurveda to treat neurological disorders and regeneration. Phytochemical assessments and spectroscopic examinations of the diethyl ether extract of methanolic concentrate of Njavara rice bran resulted in the identification of two rare flavonolignanstricins, namely 4′-O-(erythro-β guaiacylglyceryl) ether and tricin 4-O-(threo-β-guaiacylglyceryl) ether (Rao et al., 2010; Jung et al., 2014). It is now recognized that flavonoids may influence cell signaling pathways at physiological doses much below those needed to impact cellular antioxidant activity (Koch et al., 2014). Through their regulation of cell signaling pathways, flavonoids may help prevent cancer by increasing phase II detoxifying enzyme activity, decreasing proliferation, and triggering apoptosis. When human participants were fed pigmented black rice high in flavonoids, serum polyphenols and flavonoids levels were shown to be enhanced compared to baseline values (Vitalini et al., 2020). Bioactive compounds found in cereals possess antioxidative properties, which in turn counter various diseases such as heart disorders, cancer, aging, and inflammatory diseases (Espitia-Hernández et al., 2020). According to Ghasemzadeh et al. (2018), the free fraction of black rice bran had a higher IC50 value than the free fractions of red and white rice bran. Several phytochemicals found in pigmented rice prevent certain types of cancer (Henderson et al., 2012; Forster et al., 2013; Chatthongpisut et al., 2015). Through activation of effector genes, the p53 protein, and caspase enzymes, the proanthocyanidin-rich fraction isolated from red rice germ and bran extract may suppress cell growth and cause death in HepG2 cells (Wongjaikam et al., 2014; Upanan et al., 2019). This may provide a novel target for cancer treatments.

Shan et al. (2020) demonstrated that the bound fraction of foxtail millet bran regulates the expression of miRNAs to exert anti-multidrug resistance against colorectal cancer. Likewise, the enzymatic extraction of rice bran has anti-cancer potentiality against leukemic cell lines (Revilla et al., 2013). Additionally, blue corn and barley reduced the frequency of colon malignancies caused by 1,2-dimethylhydrazine and mammary carcinogenesis caused by N-methyl-N-nitrosourea, respectively (Reynoso-Camacho et al., 2015; Kubatka et al., 2016). Long-term consumption of an anthocyanin-rich extract from black rice on a continuous basis may aid in the stabilization of plaques in elderly apoE-deficient mice (Xia et al., 2006). Furthermore, black rice extracts act as antioxidants by suppressing cellular reactive oxygen species (ROS) and malondialdehyde (Lee et al., 2014).

Cyanidin-3-glucoside, the main anthocyanin compound of black rice, suppresses the occurrence of high-fat-diet-induced obesity (Kongthitilerd et al., 2020), increases hyperglycemia and insulin sensitivity via AMP-activated protein kinase in type 2 diabetic mice (Takikawa et al., 2010). The black rice pigment fraction significantly decreased plasma levels of soluble vascular cell adhesion molecule-1 (sVCAM-1), soluble CD40 ligand (sCD40L), and high sensitivity C-reactive protein (hs-CRP) in patients with coronary heart disease. In terms of the antidiabetic property, oryzanol has been reported to directly correlate with insulin sensitivity and adiponectin, indicating that it plays a critical role in type 2 diabetes (Ohara et al., 2009). Son et al. (2011) showed that oryzanol could control insulin production to maintain glucose homeostasis, correct liver enzymes’ activity, and decrease the risk of hyperglycemia associated with a high-fat diet. A diet intervention supplemented with 0.160% oryzanol and 0.05% ferulic acid alleviated hyperglycemia developed in a group of rats for 13 weeks utilizing a high-fat, high-fructose diet (HFFD) (Wang et al., 2015). Its nutritional quality and medicinal values have made rice unique among cereals. Therefore, some traditional rice varieties and other sources of cereal grains can now be portrayed as functional foods when we deploy novel processing technologies to retain higher antioxidant and anti-cancer properties in the final food products.

## Bioprocessing Applications to Attain Optimum Phenolic Compounds in Cereals

### Germination

Germination, also known as sprouting, softens the kernel upon imbibition and increases nutritional bioavailability. It is an efficient method of increasing bioactives, such as the phenolic content of grains and pulses (Ti et al., 2014; Xu et al., 2021). It has been found that germinated wheat and brown rice have more phenolic compounds, both free and bound, than ungerminated grains (Ti et al., 2014; Bei et al., 2018; Xu et al., 2021). Among the phenolics, the amount of bound ferulic acid and p-coumaric acid rose significantly during wheat and rice sprouting (Bei et al., 2018). In wheat, it is reported that the germination temperature and length have a beneficial effect on the accumulation of soluble phenolic acids, flavone C-glycosides, and lignans (Tomé-Sánchez et al., 2020). Germinated sweet corn, oat, and buckwheat grains also had much more phenolic compounds than ungerminated grains (Xu et al., 2009; Zhang et al., 2015; Xiang et al., 2017). Germination substantially altered the profile of phenolic compounds in naked barley, and the level of phenolic compounds rose significantly when germination was extended up to 36 h (Ge et al., 2021).

### Enzymatic Treatments

Enzymatic hydrolysis is often used to prepare cereal grains and their constituents, such as starch, protein, and bran, with the goal of enhancing their nutraceutical qualities and developing functional foods (Prabhu and Jayadeep, 2015). These enzymatic techniques are claimed to improve the phenolic content and bioavailability of phenolic compounds (Lima et al., 2018). However, it has also been found that the increase in phenolic content depends on the type of bran and enzyme application method used (Prabhu and Jayadeep, 2015). Rice extruded with thermostable α-amylase considerably increased the retention of total phenolics from 50.85 to 87.73% compared to conventionally cooked or extruded rice. Likewise, treatment of rice bran with a complex enzyme hydrolysis (glucoamylase, protease, and cellulase) substantially enhanced the total phenolics (46.24%) and flavonoids (79.13%) contents, respectively. Significant amounts of these phenolic compounds were released in the form of soluble conjugates rather than in their free state. Among the different phenolic acids and flavonoids, ferulic acid was the most abundantly produced, followed by protocatechuic acid and quercetin (Liu et al., 2017). Feruloyl esterase and pentopan have been shown to release ferulic acid from wheat bran selectively, and pretreatment of the bran with alcalase and termamyl enhanced ferulic acid production by up to 20 times (Ferri et al., 2020). Bread enriched with bioprocessed wheat bran through xylanase enzyme and yeast fermentation improved ferulic acid bio-accessibility (Amaya Villalva et al., 2018). Furthermore, wheat aleurone fractions treated with xylanase alone or in combination with feruloyl esterase improved ferulic acid bioavailability in obese mice fed a high-fat diet. Increased aleurone-released metabolites resulted in weight loss, decreased adiposity, increased fasting leptin levels, and better glucose metabolism (Pekkinen et al., 2014).

The cellulase treatment of oats liberated a significant quantity of ferulic acid in the insoluble fraction (Bei et al., 2018). The addition of commercial carbohydrases to maize flour resulted in an increase in the overall phenolic acid content, including ferulic acid (Cho et al., 2018). *In vitro* multienzymatic digestion of highland barley resulted in a more outstanding phenolic content than chemical extraction (Zhu et al., 2016). In whole rye flour, tannase treatment was found to increase the total phenolics and the amounts of ferulic, sinapic, and vanillic acids. However, phenolic acids’ bio-accessibility and transit efficiency were decreased when whole rye flour was treated with tannase compared to untreated whole rye flour (Lima et al., 2018).

### Fermentation

Fermentation has been extensively utilized in the food business to increase cereal grains’ shelf life, nutritional content, and organoleptic characteristics (Frias et al., 2005). Fermentation is also a viable technique for increasing foods’ phenolic content and bioavailability (Adebo and Gabriela Medina-Meza, 2020). Several widely eaten cereals, including rice, wheat, oat, maize, and sorghum, have been enhanced with phenolic content through fermentation (Saharan et al., 2017). For 24 h at 37°C, fermentation of the saccharified solution of extruded brown rice with co-cultures of *Lactobacillus plantarum*, *Lactobacillus fermentum*, and *Saccharomyces cerevisiae* significantly increased the content of free, conjugated, and bound phenolics and flavonoids, including bio-accessible phenolics (Khan et al., 2020). Using solid-state fermentation techniques, wheat grains from different cultivars fermented with a fungal *strain Aspergillus awamori*, significantly increased their total phenolic content (Sandhu et al., 2016). Likewise, fermentation with probiotic strains *Lactobacillus johnsonii* LA1, *Lactobacillus reuteri* SD2112, and *Lactobacillus acidophilus* LA-5 significantly increased free phenolic acids from 2.55 to 69.91 μg g^–1^ and 4.13 to 109.42 μg g^–1^ dry mass in whole grain barley and oat groat, respectively (Hole et al., 2012). In oats, fermentation with *Monascus anka* significantly increased the phenolics content, especially ferulic acid in the insoluble fraction and the vanillic acid in the soluble fraction. This research demonstrated that *Monascus anka* carbohydrate-hydrolyzing enzymes mediated the mobilization of phenolic compounds from fermented oats. Additionally, xylanase and cellulase were critical in degrading the cellular structure (Bei et al., 2018). Fermentation of germinated rye raised the amount of free phenolic acids, total phenolic compounds, and lignans by a significant amount (Katina et al., 2007). In maize, solid-state fermentation using *Thamnidium elegans* CCF 1456 was shown to be helpful for increasing total phenolics (Salar et al., 2012).

As with whole grain, fermentation of rice, wheat, and rye bran fractions increase their phenolic content and bio-accessibility. As defatted rice bran was fermented, it improved the bioavailability of phenolics by 64.4% when compared to raw bran (Chen et al., 2019). Likewise, fermentation of wheat bran with *Aspergillus* species resulted in the release of bound phenolic acids such as ferulic acid, chlorogenic acid, and syringic acid (Yin et al., 2018). Fermentation of rye bran increases the amount of total phenolics and free ferulic acid that are readily extracted (Katina et al., 2007).

## Innovative Processing Technologies to Increase the Phenolic Compounds in Cereal Bran

### Micronization and Microfluidization

In the fibrous matrix of cereals, phenolic compounds have limited access for digestion in the upper gastrointestinal tract due to their bound nature. Recently, advanced techniques like micronization (the process of decreasing the average diameter of the particles in a solid substance) and its modified version, microfluidization (high-pressure homogenization that creates very fine emulsion), have been found to be useful in overcoming this issue and improving the functional properties of cereal-based products such as wheat bran, corn bran, and rice bran by reducing particle size and modifying their microstructure (Wang et al., 2013, 2014a; Mert, 2020). It has been found that micronization retained the antioxidant levels of durum wheat kernels even after cooking (Martini et al., 2017), and wheat bran treated with microfluidization process increased the contents of surface-reactive and hydrolyzable phenolics (Wang et al., 2013). Antioxidant activities as measured by FRAP of micronized proso millet bran and buckwheat hulls were 78 and 23.33% higher than control, respectively (Zhu et al., 2014; Čukelj Mustač et al., 2020). Similarly, microfluidization treatment to corn bran considerably increased its antioxidant capacities by increasing the accessibility of phenolic compounds bound to or inside the bran matrix (Wang et al., 2014b). The bioavailability of bound phenolic compounds in cereals was enhanced when subjected to microfluidization. Mert (2020) has extensively reviewed the application of microfluidization in corn, rice, and wheat. However, the application of microfluidization in other cereals like barley, oat, rye, and sorghum remains relatively limited.

### Extrusion

Extrusion is a thermomechanical process, which changes the structural and functional properties in the extruded material due to the exposure to high temperature, pressure, and shear forces for a short period of time (Ramos-Enríquez et al., 2018; Zhang et al., 2018). These extrusion processes are useful in increasing the phenolic contents and improving their bio-accessibility in cereal-based products. It has been shown that ingestible phenolic compounds in wheat bran can be increased by optimizing the extrusion process (Ramos-Enríquez et al., 2018). Improved extrusion cooking treatment significantly increased the total bound phenolic acids of brown rice, wheat, and oat by 6.45, 8.78, and 9.10%, respectively. The observed effect depended on the cereal matrix and the sensitivity of free and bound phenolics (Zeng et al., 2016). In black rice bran, the total phenolics and anthocyanins were increased after extrusion processing, whereas these components were significantly reduced in polished and brown rice by extrusion processing (Ti et al., 2014). The bio-accessibility of phenolics was also found to be increased by 40.5% in extruded rice bran after *in vitro* digestion compared with raw rice bran (Chen et al., 2019). Studies conducted in a pig model showed that the extrusion of barley and oat improved the bio-accessibility of dietary phenolic acids compared with whole-grain barley and dehulled oat (Hole et al., 2012).

### Parboiling

Parboiling is a pre-milling hydrothermal process that comprises three main steps, soaking, steaming, and drying. It is generally used to gelatinize starch in the grains to seal fissures to improve the milling yield of cereals (Rocha-Villarreal et al., 2018; Zhu et al., 2020). Parboiling has been found that the crushed fractions of parboiled or bulgur einkorn wheat from Turkey showed minimal loss of phenolic compounds compared to crushed emmer fractions (Giambanelli et al., 2016). In rice, the effect of parboiling on the content of phenolic compounds and their preservation has been more profoundly studied compared with other cereals such as wheat, maize, and barley. Parboiled milled rice was found to have higher free and bound phenolic acids as compared to non-parboiled milled rice (Pal et al., 2018). It has also been found that parboiling allowed the partial preservation of free phenolics content in polished rice (Paiva et al., 2016). However, it is important to note that the parboiling effect on bioactive compounds varies according to the intrinsic properties of the grain and the processing conditions (Rocha-Villarreal et al., 2018). Hu et al. (2017) reported that germinated red rice parboiled for 5 and 15 min had higher total free phenolic content and antioxidant activity than non-parboiled germinated red rice (Hu et al., 2017). With the increase in parboiling time from 5 to 15 min, free p-coumaric acid increased from 0.20 to 0.67 mg/100 g. At 0, 2, and 5 min, bound vanillic (0.17–0.27 mg/100 g) and p-coumaric acid (6.56–8.59 mg/100 g) were at higher levels. These results indicated that thermal treatment deployed in parboiling disrupts the cell wall matrix of bran and endosperm, which helped release the bound phenolic compounds as free form having higher antioxidant capacity.

### Ultrasound Processing

Ultrasound technology is widely used in the food processing industry due to its low cost and improved final product quality features. In the food processing industry, ultrasound is used in the 20 kHz to 10 MHz frequency range. High-frequency ultrasound is used to study the physicochemical properties of food, such as acidity, firmness, and sugar. Conversely, low-frequency ultrasound is used to bring changes in the physical and chemical properties of food (Majid et al., 2015). Bonto et al. (2021) recently summarized the use of ultrasound in increasing the nutritional components of rice. In cereal product processing, ultrasound technology is used to improve the stability of products and to extract bioactive compounds, including polyphenols. For instance, ultrasound pre-treatment of wheat-dried distiller’s grain particles, a coproduct from the ethanol production process, increased the extraction yield of phenolic compounds by 14.29% (Izadifar, 2013). In rice grains, rapid extraction of phenolics from rice grains is achieved using ultrasound-assisted extraction (Setyaningsih et al., 2019). Ultrasonication has also activated, and deactivated enzymes related to polyphenolic compounds, affecting the nutritional quality of cereal bran products (Čukelj Mustač et al., 2020). Furthermore, ultrasound can improve the hydration during the germination process, accelerating the sprouting and enhancing the nutritional benefit (Miano et al., 2016). Indeed, ultrasound can be used to improve the extractability of the phenolic compounds from the cereal matrix, which can potentially be used for developing functional food products.

### Nixtamalization

Nixtamalization, also known as alkaline cooking, is a pretreatment used to alter the processing characteristics of corn. It is a traditional process in Mexico and Central America to convert corn to other products; however, it has been improved over the last decade. The process involves cooking corn in an oversaturated alkaline solution, typically, calcium hydroxide solution, for 30–40 min followed by steeping for 8–16 h. The cooked corn kernels, called nixtamal, are rinsed to remove excess lime and ground (Serna-Saldivar and Rooney, 2015; Niu and Hou, 2020). This technique dissolves the hemicellulose, alters its rheological properties, promotes protein bonding, reduces antinutrients and aflatoxins to enhance the dietary value of cereals (Schaarschmidt and Fauhl-Hassek, 2019; Cabrera-Ramírez et al., 2020; Kamau et al., 2020; Luzardo-Ocampo et al., 2020; Sunico et al., 2021). The nixtamalization method has also been investigated as a technology for increasing the number of phenolic chemicals in food (Salazar-López et al., 2018). While heat caused a reduction in the total phenolics, a significantly high concentration of ferulic acid was retained in the product, resulting in increased antioxidant activity (Gaxiola-Cuevas et al., 2017). The nixtamalized product called *nejayote* has been shown to have more phenolic acids and antioxidants than raw corn, which may be attributed to the hydrolysis of ester linkages that then liberated the phenolic acids and increased their bioavailability (Méndez-Lagunas et al., 2020). This technique has been applied to make flour and dough from other cereal grains aside from corn. One such study by Luzardo-Ocampo et al. (2020) demonstrated that nixtamalization improved the bio-accessibility of phenolics and flavonoids in sorghum. Among all phenolic compounds, gallic and chlorogenic acid had the most bioaccessibility. Moving forward, significant effort needs to be made to deploy the effect of nixtamalization on the antioxidant properties of cereal grains and their products in unraveling the contribution of specific phytochemicals to antioxidant potency.

### Pulsed Electric Fields

PEFs are a family of non-thermal food processing technologies that have recently transitioned from the laboratory to the food industry. It is primarily based on an electroporation process that includes the formation of holes in cellular membranes (Pérez-Andrés et al., 2018; Zhang et al., 2021). Brief electric pulses (1–100 s) generated by two high-voltage electrodes across a range of electric field strengths ranging from 0.1 to 80 kV/cm result in the reversible permeabilization of plant cells (Wang et al., 2018). These processing methods allow the manufacture of safe, high-quality food items rich in nutritional value, exceptional flavor, and long shelf life (Raso et al., 2014). PEF has been investigated for its ability to enhance the phenolic content of cereal grains. Lohani and Muthukumarappan (2016) showed that optimizing PEF at a flour-to-water ratio of 45% (w/v), a 2 kV/cm electric field strength, and an exposure time of 875 s resulted in a 24.8% increase in total phenolic compounds and a 33.9% increase in antioxidant activity in sorghum flour. Additionally, PEF enhanced the bioactives isolated from brown rice (Quagliariello et al., 2016). Brown rice extracts treated with PEF (2 kV/cm, 1,000 pulses, 64 kJ/kg) showed 50% higher DPPH levels and cytotoxic activity against colorectal cancer cells than untreated samples rice (Quagliariello et al., 2016). Currently, only a few studies have been conducted to determine the suitability of PEF for cereal grain processing. As a result, there is a lack of data on the sensory features, physicochemical impacts, and biochemical reactions of cereals treated with PEF.

### Infrared Heating

Infrared (IR) heating has grown in favor in recent years for a number of thermal food preparation procedures, including cereal grain roasting. Processed food products sustain less thermal damage due to the uniformity of IR heating and the short length of the process. As a result, the method has been used lately to enhance polyphenol and antioxidant recovery (Aboud et al., 2019; Faturachman and Indiarto, 2021). Wanyo et al. (2014) determined that IR treatment at an intensity of 2 kW/m^2^ and temperature of 40°C for 2 h effectively increased the phenolic content and antioxidant activities of rice bran and husk relative to hot air and enzymatic treatment using cellulase (Wanyo et al., 2014). Following IR exposure, the percent inhibition of DPPH from rice bran and husk rose from 88 to 92% and 91 to 93%, respectively. Heated air and cellulase treatments, on the other hand, had no effect on the samples’ antioxidant activities, which supports the hypothesis that the increase was driven by FIR radiation rather than heat or enzymatic activity. Similar findings were made by Irakli et al. (2018) in a study that employed an IR heating of 140°C for 15 min (Irakli et al., 2018). Rice bran samples showed a greater phenolic content and antioxidant activity under these optimal treatment settings. Additionally, the samples lost minimal vitamin E and incurred no change in oryzanol levels or fatty acid composition. These recent findings may serve to further justify the usage of IR as a future food processing alternative. Additional scientific research is needed to elucidate the interaction of food components exposed to IR radiation and its effect on the physicochemical properties, sensory properties, and nutritional values of food components.

### Micro-/Nano-Encapsulation

Encapsulation techniques such as micro-and nano-encapsulation have been increasingly used to improve storage stability, bioavailability, and targeted delivery of various food-bioactive compounds, including phenolic compounds (Assadpour and Jafari, 2019). Encapsulation can be achieved by using several physical, chemical, and physicochemical processes such as spray drying, melt extrusion, melt injection, fluid bed coating, emulsification, and liposome entrapment (Faridi Esfanjani et al., 2018; Kasote et al., 2018). Among these, liquid-based encapsulation (emulsion/nanoemulsion, solid lipid nanoparticles, and liposomes/nanoliposomes) is considered as one of the most promising techniques for protection and delivery of polyphenols due to its high-efficiency encapsulation, maintenance of chemical stability, and controlled release (Lu et al., 2016; Ozkan et al., 2020). Moreover, compared with micro-sized carriers, nanocapsules based on lipid formulations provide more surface area and thereby enhance solubility, improve bioavailability, and increase the controlled release of the nano-encapsulated phenolic compounds (Faridi Esfanjani et al., 2018).

Ferulic acid encapsulated in chitosan nanoparticles showed four times enhanced bioavailability in systemic circulation compared to its free form. Moreover, this also had higher antidiabetic potential with minimal toxicity than its free form (Panwar et al., 2018). Similarly, encapsulation of hydroxycinnamic acids such as ferulic, caffeic, sinapic, and coumaric acids in lipid-core nanocapsules protects and releases them in the simulated gastric fluid (Granata et al., 2018). Available literature showed that encapsulation techniques had not been effectively used to encapsulate crude cereals phenolics or their phenolic-rich fractions so far. However, grain components such as starch and phospholipids have been considerably used as encapsulating agents in the food industry. Rice−bran phospholipids were used to encapsulate quercetin in nanoliposomes, and these nanoliposomes were found to enhance the radical−scavenging and anti−angiogenic activities of quercetin (Rodriguez et al., 2019).

## Pigmented Cereals as Future Functional Foods and Nutraceuticals

Interest in nutraceuticals and functional foods continues to increase due to growing public interest and consumer demand (AlAli et al., 2021). As a response, food scientists, dietitians, and food industrialists are collaborating to create new functional food products. Pigmented cereal grains such as blue and purple wheat, red and black rice, purple, blue, red, and pink maize, black, blue, and purple barley, and black, purple, red, and lemon-yellow sorghum, all of which contain a variety of functional bioactive components such as polyphenols, anthocyanins, flavonoids contributing to higher antioxidant activity, that can be used to help prevent chronic diseases such as cancer, type 2 diabetes, and hypertension. Colored cereal grains serve as an attractive supplement or whole grain ingredient in the production of functional baked goods such as pan bread, flat bread, buns, rusk, cookies, extruded snacks, breakfast cereals such as pigmented cereal flakes, pigmented popped cereals, snack bars, non-alcoholic beverages, and porridges, as well as in the enhancement and retrofitting of traditional food products. Therefore, the positive health benefits of whole pigmented cereal grains, their bran fraction, phytochemicals, and antioxidant activity as described in this review article may be leveraged by the food industry to create novel nutraceutical cereal-based foods.

**FIGURE 2:**
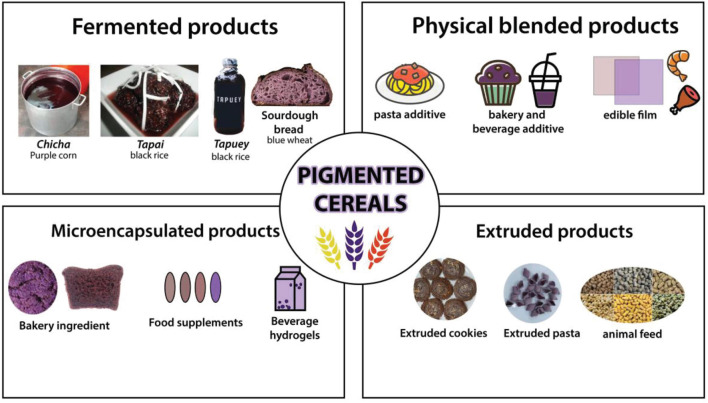
Pigmented cereal-based products.

Pigmented barley (15.3–132.3 mg Cyn-3-OGlu equiv/kg DM; Suriano et al., 2019), corn (389–7,800 mg Cyn-3-OGlu equiv/kg DM; Suriano et al., 2021), rice (79.5–473.7 mg Cyn-3-OGlu equiv/kg DM; Chen et al., 2012), sorghum (8.62–67.97 mg Cyn-3-OGlu equiv/kg DM; Dykes et al., 2009) and wheat (14.36–27.76 mg Cyn-3-OGlu equiv/kg DM; (Wang X. et al., 2020) contain a relatively higher concentration of anthocyanins and other nutrients than their non-pigmented counterpart. This has resulted in the conversion of colored cereals into a variety of functional foods with increased nutritional value and health advantages. Figure 2 illustrates a variety of food products derived from pigmented cereal grains. Other potential functional foods are shown in Table 2.

**TABLE 2:** Potential applications of food products as functional foods and nutraceuticals.

**Functional food**	**Bioactive component and bioactivity**	**Health benefit**	**References**
Bread made from Rice berry rice (Purple rice)	Anthocyanin	Improved postprandial plasma glucose and significantly increased FRAP level in healthy subjects	Anuyahong et al., 2020
Bread supplemented with black rice	Anthocyanin	Purple rice bread demonstrated lower starch hydrolysis and predicted glycemic index than Homali white rice flour bread	Thiranusornkij et al., 2019
Wheat chiffon cake supplemented with black rice	TPC and DPPH	nd	Mau et al., 2017
Black rice extract supplemented pasta	Anthocyanin, DPPH, FRAP	nd	Sethi et al., 2020
Black rice crispy rice bar	Anthocyanin	nd	Lainumngen et al., 2020
Wheat bread supplemented with stabilized rice bran	TPC, DPPH, and FRAP	nd	Irakli et al., 2015
Wheat Bread supplemented with whole grain rye flour	TPC	nd	Koletta et al., 2014
Wheat cookies supplemented with whole barley flour	Total phenolic content, MCA, DPPH, and reducing power	nd	Sharma and Gujral, 2014
Yogurt supplemented with black rice extract	TPC, C3G, P3G, DPPH, and FRAP	Purple rice extract supplemented yogurt improved plasma antioxidant capacity in healthy volunteers	Anuyahong et al., 2020
Roasted barley tea	High antioxidant activity, MCA, DPPH	Elevated lipid peroxidation inhibition in liver homogenate Increased the activity of antioxidant enzymes SOD and GSH-Px and decreased the levels of MDA and MAO in both mice liver and brain, compared to untreated mice	Omwamba et al., 2013

*DPPH, 2,2-Diphenyl-1-Picryl-Hydrazyl-Hydrate; C3G, Cyanidin-3-Glucoside; FRAP, Ferric Reducing Antioxidant Power; GSH-Px, Glutathione Peroxidase; MDA, Malondialdehyde; MAO, Manoamine Oxidase; MCA, Metal Chelating Activity; nd, Not Determined; P3G, Peonidin-3; Glucoside; SOD, Superoxide Dismutase; TPC, Total Phenolic Content.*

Fermented cereal-based meals and beverages have a higher nutritional value owing to an increase in phytochemicals and a reduction in anti-nutrients (Blandino et al., 2003; Das et al., 2011). As shown in Table 3, cereal-based products maintain a high level of phenolics. Recent research has shown the potential for fermented pigmented cereals to increase their nutritional value by boosting phytochemicals such as phenolic compounds and reducing their antinutrient content. *Chicha*, a traditional Peruvian fermented beverage made from purple maize, has been found to have enhanced phenolics, anthocyanins, and antioxidant capacity while decreasing starch digestibility (Vargas-Yana et al., 2020). A nearly 10-fold rise in gallic acid, catechin, vanillin, and resorcinol was found in pigmented barley (Bangar et al., 2021). The malting process of pigmented rice increases the phenolic acids and GABA (Santos et al., 2020). When several types of fermented rice were examined, black rice had the highest polyphenolic content and antioxidant activity, followed by red and unpigmented rice (Cai et al., 2019). Apart from its nutritional benefits, fermented black rice has good sensory qualities (Jiang et al., 2020), enhancing the product’s use when transformed into fermented snacks and beverages (dela Rosa and Medina, 2021). Pigmented cereals have also been considered in sourdough bread due to the abundance of its phytochemicals and revealed effective anti-inflammatory activities (Luti et al., 2021). Notably, fermentation conditions must be adjusted to get the highest polyphenolic content and antioxidant activity possible. For example, metabolomic analyses of fermented black rice have shown a reduction in phenolic chemicals after 60 h, which may be a result of molecular breakdown and bacterial consumption (Mu et al., 2019). While fermented colored barley, maize, and rice have been widely researched for their nutritional benefits, other pigmented cereals have received less attention. Metabolomics study of fermented pigmented grains has the potential to provide new light on the phenolic diversity and its interaction with microorganisms. Natural pigments such as anthocyanins and proanthocyanidins found in pigmented grains have been used to increase the nutritional content and prolong the shelf life of food items (Chatham et al., 2020). Due to the low price attached to broken-colored cereal, grains may be processed to create food additives (Sapna and Jayadeep, 2020). The encapsulation of phenolic compounds is used as a co-ingredient in bread goods (Papillo et al., 2018; Thiranusornkij et al., 2019), food supplements (Norkaew et al., 2019), beverage hydrogels (Guo et al., 2018), and probiotic products.

**TABLE 3:** Cereal products that retain a higher amount of polyphenolic compounds.

**Cereal products**	**Bioactive compounds and bioactivity**	**Retention of bioactive compounds**	**References**
Cereal bran enriched ready to eat breakfast cereal porridge	TPC	Rice bran enriched porridge recorded the highest total phenolic content (0.97 mg GAE/g) followed by wheat and oat bran enriched product. As the bran supplementation increased from 5 to 15%, the total phenolic content increased from 0.65 to 1.02 mg GAE/g.	Sharma et al., 2016
Multi-whole grain mix for drink and porridge	TPC, TAA	The 100 g of the mix had nutraceuticals like carotenoids (290 μg), gamma-tocopherol (4.6 mg), alpha-tocopherol (1.5 mg), and polyphenols-soluble, bound and total (94, 132, and 226 mg GAE). Bioactive properties like vitamin E, free radical scavenging, and total antioxidant activity were 2.6 IU, 153 mg CAE/100 g, and 17 mg tocopherol equivalent, respectively.	Itagi et al., 2012
Ready-to-eat flakes from cereals like maize grits, pearled barley, hulled oats, wheat, pearl millet, and sorghum	TPC, DPPH activity	Blistered cereal flakes are excellent ready-to-eat snacks, as they are rich in total polyphenols (16-58 mg GAE/100 g) and exhibit high antioxidant activity	
Tortillas from whole pigmented extruded Mexican maize flours	TPC, TAC, and total hydrophilic antioxidant content	Tortillas elaborated from extruded pigmented Mexican maize flour retained 76.4–87.5%, 27.1–65.4%, and 87.2–90.7%, respectively, of the total phenolics, anthocyanins, and total hydrophilic antioxidant content present in raw grains. The blue maize extruded products were the best in overall polyphenolic and antioxidant content, followed by red, white, and yellow maize	Aguayo-Rojas et al., 2012
Pigmented corn tortilla and tortilla chip	Free and soluble conjugated ferulic acid	Lime-cooking, tortilla baking, and tortilla chip frying increased the amount of free and soluble conjugated ferulic acid	de la Parra et al., 2007
Gluten-free maize noodles	TPC	Flint maize noodles retained 50 and 66% phenolics by traditional and ecological nixtamalization process	Das et al., 2017
Wheat-purple rice biscuits	TPC, TFC, Anthocyanin, DPPH, ABTS activity	Increasing the purple rice resulted in higher antioxidant properties compared to the wheat flour biscuits	Klunklin and Savage, 2018
Black rice chiffon cake	TPC, TAC, and DPPH activity	Total phenols, anthocyanins, and scavenging ability of baked cake extracts increased with increased black rice powder levels from 10 to 100%	Mau et al., 2017
Popped black rice and beaten black rice	TAC and DPPH activity	Popped black rice and beaten black rice showed higher anthocyanin compared to white rice. Popped and boiled rice of Mamihunger, black rice displayed higher DPPH-antioxidant activity (88.74 and 84.74%, respectively) compared to puffed and Beaten rice products of other rice varieties.	Pal et al., 2019

*ABTS, 2,2′-azino-bis (3-ethylbenzothiazoline-6-sulfonic acid); DPPH, 2,2-diphenyl-1-picryl-hydrazyl-hydrate; APF, All-purpose flour; CAE, Catechin Equivalent; GAE, Gallic Acid Equivalent; TAC, Total Anthocyanin Content; TAA, Total Antioxidant Assay; TFC, Total Flavonoid Content; TPC, Total Phenolic Content.*

Physical mixing of cereal grains rich in phenolic compounds with polymers and food matrix resulted in various product additives and biopolymers (Tiozon et al., 2021). The addition of red sorghum to pasta enhanced the amount of free phenolic acids and resistant starch (Khan et al., 2013). Meanwhile, the inclusion of black rice extracts extends the shelf life of muffins and improves their eating quality (Croitoru et al., 2018). Combining pigmented grain starch has a twofold purpose: starch interacts with other polymers to increase their hydrophilicity, and its phenolic chemicals provide antioxidant qualities. When black rice extracts are combined with a chitosan-starch film, the antioxidant and light barrier characteristics are enhanced (Yong et al., 2019). Additionally, purple corn extracts containing chitosan and silver nanoparticles improved the film’s pH sensitivity and antibacterial activity (Qin et al., 2019). The resultant nanocomposite film based on pigmented grains has been shown to monitor the deterioration of pork (Yong et al., 2019) and seafood items (Ge et al., 2020). The change in pasting, thermal, and rheological characteristics is due to the complexation of phenolic chemicals with starch and other polymers (Zhu, 2015).

Extruded pigmented cereals have gained significant attention due to their nutritive value and ease of consumption. By and large, the overall phenolic content is decreased, but their bioavailability is increased (Hole et al., 2013). For instance, extruded puffed rice increased extractable phenolic acids, namely gallic acid and protocatechuic acid, but significantly decreased cyanidin-3-glucoside (Bagchi et al., 2021). In contrast, extruded blue corn showed cyanidin-3-glucoside stability and retention, which may be related to previous nixtamalization (Escalante-Aburto et al., 2013). Enzymes may be used to enhance the retention of phenolics in extruded goods (Zeng et al., 2018). Additionally, as shown with extruded sorghum, controlling factors such as feed moisture and extrusion zone temperature may avoid additional phenolic loss (Ortiz-Cruz et al., 2020). Other components can be added in cereals like vegetable (Xu et al., 2021) and fruit (Bhat et al., 2019) extracts. Extrusion-based three-dimensional technology has emerged as a new mode to diversify products without sacrificing their nutritional or sensory qualities (Prabha et al., 2021).

## Conclusion

In general, promoting wholegrain cereal grain consumption over milled endosperm to prevent non-communicable diseases is widely accepted. However, owing to rancidity, poor shelf-life, and reduced palatability, different processing methods are being used to produce milled goods for broader consumption. As a consequence, we lose the nutritional density found in grains, such as rice bran. The enormous genetic diversity that exists for enriched phenolic chemicals and flavonoids in cereals as purple, variable purple, and red cereal grains has provided new possibilities to market them as future functional food. However, it is critical to evaluate the final concentrations of these free phenolic compounds in pigmented food items after cooking/baking to guarantee bioavailability of these phytochemicals and food safety concerns to remove aflatoxins mycotoxins, and pesticides to translate human health benefits. Identifying the optimal post-harvest processing techniques to maintain greater polyphenolic compounds via traditional cooking and baking processes should be encouraged to preserve the human health advantages. More than 150 million tons of wheat bran and 76 million tons of rice bran nutritional material are produced as a byproduct of milling and are mostly discarded. It can be channeled into technological advancements in processing technologies such as micronization, microfluidization, ultrasound processing, nixtamalization, pulsed electric fields, and micro/nano-encapsulation to extract bioactives and phenolic compounds and produce various functional byproducts with enormous human health benefits.

Furthermore, bioprocessing techniques to produce different functional food products from germination sprouts, fermentation and enzymatic treatments of bran and pigmented grains to enhance the bioaccessibility of phenolic compounds in producing distinct functional byproducts may be used. Implementing holistic strategies to identify donor lines with enriched bioactives using untargeted metabolite profiling to identify novel phenolic compounds with higher antioxidant potential and deploying state-of-the-art processing applications to produce final food products/by-products with higher bioactives will be beneficial in creating novel opportunities to position cereals as staple foods to meet human nutrition needs. Long-term clinical studies for the whole range of functional food items produced from cereals are required to determine long-term benefits. Additional research on the gut-microbiome health advantages of colored grains is needed. We would like to highlight that the consumption of pigmented cereals and their derived functional foods as staples by definition means that improving staples-based diets can deliver maximum benefits, particularly to the poorer sections of society, while also satisfying the urban consumers’ health concerns.

## Author Contributions

NS conceptualized the review article and edited the article with inputs from DK, RT, KS, HI, PR, AK, and AR. All authors contributed to the article and approved the submitted version.

## Conflict of Interest

The authors declare that the research was conducted in the absence of any commercial or financial relationships that could be construed as a potential conflict of interest.

## Publisher’s Note

All claims expressed in this article are solely those of the authors and do not necessarily represent those of their affiliated organizations, or those of the publisher, the editors and the reviewers. Any product that may be evaluated in this article, or claim that may be made by its manufacturer, is not guaranteed or endorsed by the publisher.

## Abbreviations

Values are % change from first to last data point, lowest to highest concentration or control vs. variables:

ABTS: 2,2′-azino-bis (3-ethylbenzothiazoline-6-sulfonic acid)
AE: Aqueous Extract
DPPH: 2,2-diphenyl-1-picryl-hydrazyl-hydrate
B: Bound
BCR: Black Colored Rice
EE: Ethanolic Extracts
ENCF: Extruded Nixtamalized Corn Flour
F: Free
FRAP: Ferric reducing antioxidant power
UGAL2: *L. lactis* ssp. *Lactis*
UGAL1: *W. confusa*
15GAL: *L. plantarum*
16GAL: *L. brevis; L. plantarum*
DI-PROX MTTX: *L. brevis*
LH-B02: *L. helveticus*
LAF-4: *K. Marxianus* subsp. Marxianus
ND: Not Detected
RCR: Red Colored Rice
RH: Relative Humidity
RPM: Revolutions Per Minute
SC: Soluble Conjugate
T: Total
TAC: Total Anthocyanin Content
TFC: Total Flavonoid Content
TPC: Total Phenolic Content
UI: Ultrasonic Intensity
WR: White Rice.
