# Supplementary material for: Food Processing Technologies to Develop Functional Foods With Enriched Bioactive Phenolic Compounds in Cereals
Source: Front Plant Sci. 2021 Nov 30;12:771276. doi: 10.3389/fpls.2021.771276 (PMC8670417; doi:10.3389/fpls.2021.771276)
Supplement: Supplementary file 1 [file Table_1.pdf]

Supplementary Table 1. Phenolic compounds and antioxidant activities as affected by various pre-treatment methods

**Processing/ postharvest method/s applied**	**Botanical Source**	**Fraction/s**	**Treatment details**	**TPC**	**TFC**	**TAC**	**FRAP**	**DPPH**	**ABTS**	**ORAC**	**References**
Extrusion	Rice	Bran	300 RPM screw speed	↓23%F; ↓50.7%B; ↓36.3%T	-	-	-	-	-	↑154.5%F; ↑47.5%B	(Chen et al., 2019)
	Wheat	Bran	100 RPM screw speed	↑3.01%F; ↑61.44%B; ↑42.95%T	-	-	-	↓4.76%F; ↑22.50%B; ↑25.74%T	↓15.60 %F; ↑43.66%B; ↑35.37%T	-	(Ramos-Enríquez et al., 2018)
	Sorghum	Bran	100 RPM screw speed	↑22.40%F	-	-	-	↑24.48%F	-	-	(Ortiz-Cruz et al., 2020)
	Rice	Whole grain	120 °C with 26.6 RPM screw speed	↓76.18%F ↑4.47%B	-	-	-	-	↓63.06%F; ↑6.66%B	-	(Zeng et al., 2016)
	Wheat			↓38.63%F; ↑ 4.20%B	-	-	-	-	↓36.55%F; ↑1.64%B	-	
	Oat			↓27.16%F; ↑15.60%B	-	-	-	-	↓14.39%F; ↑8.18%B	-	
Germination	Rice	Whole grain	Up to 48 h at darkness at 20°C with 99% RH	↑63.2%T; ↑76.67%F; ↑44.64%B	↑23.62%T ↑23.40%F ↑23.63%B	-	↑22.92%T ↑19.0% F ↑28.19%B	-	-	↑87.86%T ↑64.96%F ↑163.04% B	(Ti et al., 2014)
	Wheat	Whole grain	Up to 96 h at 25 ◦C	-	-	-	-	-	-	↑311.52%F ↑81.44%B	(Kim et al., 2018)
	Barley	Whole grain	24±1 h at 24°C	-	-	-	-	-	-	↑81.18%T	(Kruma et al., 2016)
	Wheat	Whole grain	Up to 7 days at darkness at 12–21°C with >90% RH	↑271.02%F	-	-	↑45.45%F	↑64.10%F	↑181.42%F	↑164.53%F	(Tomé-Sánchez et al., 2020)
	Corn	Whole grain	Light: 24-h photoperiod, irradiance of 200 μmol·m^{-2}s^{-1}) at 20°C	↑150%F; 100%B	↑141.82%T	-	-	-	-	↑100%T ↑296.13%F ↓49.47%B	(Xiang et al., 2017)
			Dark: 24-h in darkness at 20°C	-	↑100%T; ↑100%F; ↓19.30%B	-	-	-	-	↑218.58%F	
Thermal and	Sorghum	Whole	Up to 48 h at	↑5.36%T	↓37.08%T	-	-	-	-	-	(Hithamani and

hydrothermal		grain	25ºC								Srinivasan, 2014)
			Boiling for 10 mins (open pan)	↓42.86%	↓46.07%T	-	-	-	-	-	
			Sand roasting (150ºC for 5min)	↑49.11%T	↓26.97%T	-	-	-	-	-	
			Pressure cooking (15 psi for 15 mins)	↓50.89%T	↓51.69%T	-	-	-	-	-	
Microwave			450W for 4 mins	↓46.43%T	↓51.69%T	-	-	-	-		
Fermentation	Wheat	Whole grain	5ml inoculum *Lactobacillus rhamnosus* A71 and *Saccharomyces cerevisiae* at 30ºC for 24 h	↑27.78%T (*L. rhamnosus*); ↑13.58%T (*S. cerevisiae)*	-	-	↑24.36%T (*L. rhamnosus*); ↑75.27%T (*S. cerevisiae)*	-	-	-	(Dordević et al., 2010)
	Rye			↑39.39%T (*L. rhamnosus*); ↑14.20%T (*S. cerevisiae)*	-	-	↑55.93%T (*L. rhamnosus*); ↑19.46%T (*S. cerevisiae)*	↓1.85%T (*L. rhamnosus*)	-	-	
	Buckwheat			↑14.16%T (*L. rhamnosus*); ↑4.93%T (*S. cerevisiae)*	-	-	↑4.27%T (*L. rhamnosus*); ↑0.67%T (*S. cerevisiae)*	↓17.34%T (*L. rhamnosus*); ↓13.19%T (*S. cerevisiae)*	-	-	
	Barley			↑22.56%T (*L. rhamnosus*); ↑12.80%T (*S. cerevisiae)*	-	-	↑28.53%T (*L. rhamnosus*); ↓6.71%T (*S. cerevisiae)*	-	-	-	
	Oat	Bran	6% (w/w) of compressed baker's yeast and fermented for 6 h at 30 °C	↓19.36%F; ↑26.12B; ↑16.87%T	-	-	-	↑11.06%F; ↑3.95B; ↑6.12%T	-	-	(Özkaya et al., 2017)
Hydrothermal			Autoclave at 121°C for 1.5h, pH 4.0	↓18.60%F; ↑53.13B; ↑38.53%T	-	-	-	↑22.85%F; ↑11.47B; ↑14.94%T	-	-	
Fermentation	Rye	Whole grain	3-5 x108 UFC/100 g	Sourdough: ↑48.99%T(LH-	-	-	-	Sourdough: ↑6.02%T	Sourdough: ↑30.36%T(LH-	-	(Banu et al., 2010)

			dough 40°C for 16 h with	B02+LAF-4); ↑117.93%T (DI-PROXMTTX); ↑68.87T (15GAL+16GAL); ↑38.25%T(UGAL1); ↑15.82%T (UGAL2)				(LH-B02+LAF-4); ↑ 43.61%T(DI-PROX MTTX); ↑26.32T(15GAL+16GAL); ↑4.51%T(UGAL1); ↑ 5.26%T (UGAL2)	B02+LAF-4); ↑ 80.36%T(DI-PROX MTTX); ↑105.36%T(15GAL+16GAL); ↑64.29%T(UGAL1; ↑ 53.57%T (UGAL2)		
Enzymatic treatment	Oats	Whole grain	0 To 5.43 U/g cellulase From *A. niger* at 30 °C for 12 hours	↑41.30%F; ↓48.24B	-	-	-	-	-	-	(Bei et al., 2018)
	Rice	Bran	Cellulase at 50°C for 90 mins.	↑10%T(WR), 17%T(BCR),12.95%F(WR), 47.56%F(BCR), 6.67%B (WR), 5.72%B(BCR)	↑4.93%T (WR), 25.75%T (BCR) (cellulase)	-	-	↑18%T(WR), 21%T(BCR)	-	-	(Prabhu and Jayadeep, 2015)
			Xylanase at 50°C for 90 mins.	↑14%T(WR), 20%T(BCR),9.35%F(WR), 49.75%F(BCR), 19.61%B (WR), 9.44%B(BCR)	↑2.73 (WR),18.32T(BCR) (xylanase)	-	-	↑13%T(WR), 15%T(BCR)	-	-	
			Mixture	↑40%T(WR), 21%T(BCR),16.55%F (WR), 58.19%F(BCR), 11.76%B (WR), 7.99%B(BCR)	↑12.31%T(WR), 37.89%T(BCR)	-	-	↑29%T(WR), 41%T(BCR)	-	-	
Enzymatic treatment	Wheat	Bran	0.015g cellulase at 50 °C for 1	↓68.67%T	↓74.51%T	-	-	↓42.25%T	↓51.29%T	-	(Wang et al., 2019)
	Wheat	Bran	Cellulase followed by steaming for 40 mins.	↓64.45%T	↓73.54%T	-	-	↓31.02%T	↓38.51%T	-	
	Wheat	Bran	Cellulase followed by roasting 180 °C	↑21.93%T	↓23.25%T	-	-	↓11.40%T	↓0.32%T	-	

			for 15 mins.								
Enzymatic treatment	Rye	Whole grain	1000U/kg tannase at 37°C for 120 mins	-	-	-	↑61%T	↑1150%T	-	-	(Lima et al., 2018)
	Rice	Bran	Enzyme mixture for 190 min at 57.5°C	↑46.34%T; ↑117.21%F; ↑33.35%SC	↑79.13%T; ↑69.27%F; ↑96.09%SC		↑159.14%T; ↑153.27%F; ↑163.53%SC			↑49.99%T; ↑83.30%F; ↑30.35%SC	(Liu et al., 2017)
	Wheat	Bran	Enzyme mixture at 60°C for 1 h	↑63.83%T	-	-	-	-	-	-	(Ferri et al., 2020)
	Corn	Whole grain	β-glucanase	↑22.21%T	-	-	-	↑50.52%T	↑62.05%T	-	(Cho et al., 2018)
	Corn	Whole grain	Pentopan 500 BG (Endo 1-4 β-xylanase)	↑60.83%T	-	-	-	↑27.32%T	↑22.91%T	-	(Cho et al., 2018)
	Barley	Whole grain	Multi-enzymatic digestion	-	-	-	-	-	-	↑152.47%T	(Zhu et al., 2016)
Fermentation	Oats	Whole grain	10% (v/ v) *M. anka* spore suspension at 30 °C for 14 days	↑1585.8%F; ↑142.71%B	-	-	-	-	-	-	(Bei et al., 2018)
Enzymatic followed by fermentation			Cellulase at 4.45 U/g followed by 10% (v/ v) fermentation	↑3036.56%F; ↑243.49%B	-	-	-	↑2.05x103%F; ↑200%B	-	-	
Fermentation followed by ultrasound	Wheat	Bran	*S. cerevisiae* (107 CFU/ml) at 30°C for up to 6 days and ultrasound at 40 °C for 1h	↑112%T	-	-	-	-	-	-	(Călinoiu et al., 2019)
	Oat			↑83%T	-	-	-	-	-	-	
	Sorghum	Whole grain	Whole grain sorghum flour mixed with sterile distilled water (27°C for	↑4.34%T (27°C for 24 h vs 27°C for 48 h); ↑4.34%T (27°C for 24 h vs 27°C	↑45.93%T (27°C for 24 h vs 27°C for 48 h); ↑30.73%T (27°C for 24 h	-	-	-	↑0.52%T (27°C for 24 h vs 27°C for 48 h); ↑3.19%T (27°C for 24 h vs 27°C	-	(Adebo et al., 2018)

Fermentation			24 h) (1:1, w/v)	for 72 h)	vs 27°C for 72 h)				for 72 h)		
			L. Fermentum FUA 3165 and/or *L. Fermentum* FUA 3321 105 CFU/ml for 72 h at 28°C for high Tannin Type	↓30.09%T (3165); ↓31.92%T (3321); ↓24.30% T (3165+3321)	↓35.44%T (3165); ↓37.74%T (3321); ↓10.91% T (3165+3321)	-	-	-	↑90.57%T (3165); ↑110.51%T (3321); ↑100% T (3165+3321)	-	
			L. Fermentum FUA 3165 and/or *L. Fermentum* FUA 3321 105 CFU/ml for 24 h at 34°C low Tannin Type	↓43.67%T (3165); ↓47.61%T (3321); ↓41.80 %T (3165+3321)	↓19.347%T (3165); ↓22.13%T (3321); ↓10.13 %T (3165+3321)	-	-	-	↑312.71%T(3165) ; ↑322.88%T(3321) ; ↑308.47%T (3165+3321)	-	
Fermentation and enzymatic treatment	Wheat	Bran	0.05% (w/ w) xylanase and 0.27% (w/w) baker's yeast	↑883.38T; ↑31.84%F; ↑61.10% B	-	-	-	-	↑176.34%T; ↓9.09%F; ↑201.22%B	-	(Amaya Villalva et al., 2018)
Fermentation, enzymatic treatment followed by baking			Baking at 10 min at 240°C	↑45.22%T; ↑17.76%F; ↑76%B	-	-	-	-	↑119.75%T; ↓4.55%F; ↑159.42%%	-	
Fermentation (solid-state)	Corn	Whole grains	Spore suspension of *T. Elegans.* for up to 5 days	↑16.82%T	-	-	-	↑51.90%T	↑58.06%T	-	(Salar et al., 2012)
	Barley	Whole grains	200 µl (*L. johnsonii LA1*, *L reuteri SD2112, and L. acidophilus LA-5*)	↑2641.57%F (*LA1*) ↑2088.24%F (*SD2112*) ↑1783.14%F (*LA-5*) ↑21.32%B (*SD2112*) ↑2062%B (*LA-5*)	-	-	-	-	-	-	(Hole et al., 2012)

	Oat	Whole grains	200 µl (*L. johnsonii LA1*, *L. reuteri SD2112, and L. acidophilus LA-5*)	↑2549.39%F (*LA1*) ↑2220.82%F (*SD2112*) ↑2061.26%F (*LA-5*) ↓9.55%B (*SD2112*) ↓10.10%B (*LA-5*)	-	-	-	-	-	-	(Hole et al., 2012)
Fermentation	Wheat	Whole grains	100-µl *Bifidobacterium spp., B. Animalis, B. Breve, and B. Longum* at 37°C for 24h	↑480.00%T	-	-	-	↑3.03%T	↑4.23%T	-	(Ayyash et al., 2018)
	Millet	Whole grains	1 ml spore suspension of *Aspergillus awamori* at 30 °C up to 10 days.	↑372.89%T	-	-	-	↑3.75%T	↑2.12%T	-	(Salar et al., 2016)
	Rye	Whole meal	*S. cerevisiae*, *L. casei*, *L. brevis*, *S. Chevalieri yeast* at 60 min at 35°C and 85% RH	-	-	-	-	↑82.32%F	-	↑159.39%F	(Skrajda-Brdak et al., 2019)
	Rice	Bran	*R. oryzae* at 1x104 spores/g bran at 30°C for 5 days	↓99.4%F; ↓40%B; ↓71.6%T	-	-	-	-	-	↑141.8%F; ↑45.3%B	(Chen et al., 2019)
	Wheat	Bran	*S. cerevisiae* and 0.1g LAB starter powder (*L. bulgaricus* and *S. Thermophiles*) at 37°C for 16 h	↓26.03%T(yeast); ↓22.82%T(LAB); ↓15.03%T (yeast + LAB)	-	-	-	-	-	-	(Zhao et al., 2018)
Fermentation	Millet	Whole	Natural	↑62.80%F;	-	-	-	-	-	-	(Gabaza et al.,

		grain	microflora for 24-36 hours at 23-25°C	↓30.76%B; ↓ 20.71%T							2016)
Fermentation followed by cooking			15-20 mins with 300 ml water	↑9.02%T (Raw vs Cooked); ↑47.66%T (Fermented vs Cooked);	-	-	-	-	-	-	
Parboiling	Rice	Dehusked and polished grains	Heated in water at 65°C for 300 min and autoclaved at 116°C10 min.	↓32.76%	-	-	-	↓16.66%F	-	-	(Walter et al., 2013)
	Millet	Whole grain	Soak-boil method for 5 mins and air-dried for 24-48 h	↑63.87 to 199.20%F; ↑40.89 to 46.73%B	-	-	-	↑110.54%F; ↑32.27%B	-	-	(Bora et al., 2019)
	Rice	Dehusked and polished grains	Water bath at 60°C for 4 h and autoclaved for 10 min at 108°C	-	-	-	-	↑369.62%F; ↑20.83%B	↑322.35%F; ↑492.31%B	-	(Paiva et al., 2016)
Hydrothermal	Sorghum,	Whole meal	Boiling for 21mins for fonio, 15 mins for Millet, and 12 mins for sorghum	↓48.58%F; ↓44.57%B; ↓45.17%T	-	-	-	-	↓75.94%F; ↓38.06%B; ↓51.69%T	-	(N'Dri et al., 2013)
	Fonio			↓17.48%F; ↓3.46%B; ↓7.46%T	-	-	-	-	↓90.21%F; ↓43.49%B; ↓59.67%T	-	
	Millet			↓14.42%F; ↓53.76%B; ↓45.83%T	-	-	-	-	↓68.45%F; ↓21.44%B; ↓38.86%T	-	
	Rice	Whole grains	20 ml extracts extracted with 60, 80, or 100 °C in a water bath at 30 min intervals, over 0-120 min	↓4.5%-7.3%T (120 mins)	↓1.1%-4.6%T (120 mins)	-	↑0.82%T(60°C , 120 mins) ↑1.63%T(80°C , 120 mins) ↑4.08%T(100°C, 120 mins)	↓3.21%T (60°C, 120 mins) ↓4.36%T (80°C, 120 mins) ↓8.41%T (100°C, 120 mins)	↓5.75%T (60°C, 120 mins) ↓4.51%T (80°C, 120 mins) ↓1.61%T(100°C, 120 mins)	-	(Zeng et al., 2019)
Infrared	Rice	Bran	Heating at 140°C for 15	↑6.79%F; ↑22.38%B	-	-		↓39.17%F; ↑36.81%B	-	-	(Irakli et al., 2018)

			min								
Thermal	Rice	Bran	Heated in an oven at 150◦C for 40 min	↓16.53%F; ↓9.94%B; ↓15.15%T	-	-	↓0.45%F; ↑50.00%B; ↓13.11%T	↓13.68%F; ↓12.13%B; ↓13.19%T	↓13.85%F; ↑136.45%B; ↑23.97%T	-	(Irakli et al., 2020)
Infrared			Infrared at 40◦C for min	↓14.02%F; ↓11.11%B; ↓13.35%T	-	-	↑7.87%F; ↑3.66%B; ↑6.61%T	↓12.39%F; ↓20.22%B; ↓14.34%T	↓12.29%F; ↓5.61%B; ↓11.87%T	-	
Microwave			Microwave for 2 min at 650 W	↓11.35%F; ↓8.33%B; ↓10.65%T	-	-	↓24.21%F; ↑61.79%B; ↓0.99%T	↓11.61%F; ↓2.57%B; ↓7.84%T	↓7.60%F; ↑143.55%B; ↓34.20%T	-	
Thermal	Rice	Bran and husk	120°C for 30 min using hot-air oven	↑1.70%T (Bran); ↑0.89%T (Husk)	↓20.62%T (Bran); ↓22.93%T (Husk)		↑1.05%T (Bran); ↑4.07%T (Husk)	↓0.80%T (Bran); ↓14.71%T (Husk)	-	-	(Wanyo et al., 2014)
Infrared			40°C for 2h	↑15.06%T(Bran); ↑96.43%T (Husk)	↓7.47%T(Bran); ↓13.91%T (Husk)		↑20.44%T(Bran); ↑35.21%T (Husk)	↑4.87%T(Bran); ↑0.75%T (Husk)	-	-	
Enzymatic treatments			Cellulase reaction for 24 h at pH=5, 50°C	↓13.35%T(Bran); ↑10.71%T (Husk)	↓4.12%T(Bran); ↑5.26%T (Husk)		↑0.35%T(Bran); ↑1.70%T (Husk)	↑0.52%T(Bran); ↑1.48%T (Husk)	-	-	
Thermal followed by ultrasound	Wheat Oat	Bran	10 min, 80◦C processing and ultrasonic bath at 40◦C for 1h	↑22.49%T(Wheat) ↑25.84%T(Oat)	-	-	-	-	-	-	(Călinoiu and Vodnar, 2020)
Thermal, hydrothermal and fermentation (solid- state)	Sorghum	Whole grain	Boiling for 30 mins, fermentation (LAB) for 8h at 37°C); Steaming (at Steaming vessel for 30 mins); Flaking (400 to 600 RPM)	↓30.78%T(boiling); ↓21.95%T (fermented); ↓35.77%T (fermented and steamed); ↓29.73%T (fermented, steamed, and flaked)	-	-	-	↓2.41%T(boiling); ↑41.02%T (fermented); ↓52.79%T (fermented and steamed); ↓29.32%T (fermented, steamed, and flaked)		-	
Thermal	Rice	Whole grain	Superheated steam for 30 mins.	↑36.88%T	-	-	-	-	-	-	(Xu et al., 2015)
Extrusion			Screw speed of 100 RPM and	↑1.71% to 8.67%T	-	-	-	-	-	-	

			Feed rate of 1.5 kg/h)								
Enzymatic treatment			α-amylase at 0.1%, db for 12 hours at 4 °C	↑24.52%T	-	-	-	↑25.57%T	↑42.48%T	-	
Thermal	Barley	Whole grain	Traditional sand roaster at 280± 5°c for 20 secs	↓8.5 To 32.9%T	↓24.5 To 51.1%T	-		↑38.1 to 108.2%T	-	-	(Sharma and Gujral, 2011)
Microwave			Microwave for 120 secs at 900 w	↓24.4 to 43.1%T	↓25.9 To 53.2%T	-		↑16.8 to 80.2%T	-	-	
Thermal	Barley	Bran	Steam explosion at 4.0 MPA at 220 °C	↑571.49%F (220 °C); ↑184.43%SC (220 °C)	-	-	↑181.38%T (220 °C for 120 sec)	-	↑815.03%T (220 °C for 120 sec)	-	(Gong et al., 2012)
	Teff	Whole grain	Boiling for 2, 3.5 and 5 min;	↓18.65%T (2 mins); ↓20.24%T (3.5 mins); ↓24.21%T (5 mins);	↓23.57%T (2 mins); ↓27.18%T (3.5 mins);↑15.33%T (5 mins);	-	↓16.15%T (2 mins); ↓19.15%T (3.5 mins); ↓19.63%T (5 mins);	↓12.75%T (2 mins); ↑3.34%T (3.5 mins); ↓0.40%T (5 mins);	↓55.15%T (2 mins); ↓54.78%T (3.5 mins); ↓59.56%T (5 mins);	-	(Kataria et al., 2021)
			Roasting for 180 ±20°C for 5, 7.5 and 10 min;	↑0.40%T (5 mins); ↓2.38%T (7.5 mins); ↑0.79%T (10 mins);	↑8.65%T (5 mins); ↑7.77%T (7.5 mins); ↓0.14%T (10 mins);	-	↓15.47%T (5 mins); ↓14.60%T (7.5 mins); ↓27.47%T (10 mins);	↓7.99%T (5 mins); ↓11.01%T (7.5 mins); ↓13.55%T (10 mins);	↓41.15%T (5 mins); ↓39.34%T (7.5 mins); ↓37.13%T (10 mins);	-	
Microwave			Microwave processing (900W) for 2, 3.5 and 5 mins;	↑0.79%T (2 mins); ↑9.92%T (3.5 mins); ↑24.60%T (5 mins);	↓0.95%T (2 mins); ↑10.15%T (3.5 mins);↑4.29%T (5 mins);	-	↓21.66%T (2 mins); ↓15.18%T (3.5 mins); ↓8.32%T (5 mins);	↓9.37%T (2 mins); ↓8.32%T (3.5 mins); ↓4.98%T (5 mins);	↓38.97%T (2 mins); ↓30.88%T (3.5 mins); ↑6.62%T (5 mins);	-	
Hydrothermal			Autoclave at 121°C at 15psi for 120 mins	↓57.14%T	↓23.77%T	-	↓27.37%T	↓11.74%T	↓41.91%T	-	
	Oat	Whole grain	600 w at 90 °c	↑2.58%T; ↑2.89%F; ↑11.45%SC; ↓10.76%B	-	-	-	-	-	↑8.18%T (90 °C)	(Chen et al., 2018)

Ultrasound	Corn	Whole grain	50% amplitude level at 6°C for 30 mins	↑3418.62%T	-	↑542.69% T	-	-	-	-	(Muangrat et al., 2017)
	Rye	Bran	45 kHz for 29 mins at 66 ◦C	↑40%T	↑32.63%T	-	-	-	-	-	(Iftikhar et al., 2020)
	Rice	Whole grain	45 °C, 25 min, cycle 0.4 s−1 amplitude 47%	↑111.21%T(RCR); ↑450.84%T(BCR);	-	-	-	-	-	-	(Setyaningsih et al., 2019)
	Sorghum	Whole grain	0.24–0.40 W cm-2 at 5 To 75 mins	↑2.46%T(0.24 vs.0.32UI); ↑5.65%T(0.24 vs.0.40UI); ↑3.11%T(0.32 vs.0.40UI)	↑9.52%T(0.24 vs.0.32UI); ↑9.77%T(0.24 vs.0.40UI); ↑0.22%T(0.32 vs.0.40UI)	-	-	-	-	-	(Hou et al., 2016)
	Sorghum	Whole grain	T1(amplitude: 40%, Time: 5 min); T2(amplitude: 60%, Time: 5 min); T3 (amplitude: 40%, Time: 10 min); T4 (amplitude: 60%, Time: 10 min)	↑6.78%T(T1); ↑3.39%T(T2); = (T3); ↓4.24%T(T4)	↑15.91%T(T1); ↑3.41%T(T2); ↓6.82%T(T3); ↓11.36%T(T4)	-	↑6.90%T(T1); =T(T2); ↓6.90%T(T3); ↓10.34%T(T4)	↑6.39%T(T1); ↑6.09%T (T2); ↓3.01%T(T3); ↓6.71%T(T4)	-	↑6.62%T(T1); ↑1.02%T (T2); ↓8.94%T(T3); ↓5.95%T(T4)	(Hassan et al., 2020)
Pulsed electric Field	Sorghum	Whole grain	2 KV /cm EFI and 875 µs	↑24.8%T	-	-	-	↑33.9%T	-	-	(Lohani and Muthukumarappan, 2016)
	Rice	Whole grain	2 KV/cm,1000 pulses, 64kj/kg	-	-	-	-	↑50%T	-	-	(Quagliariello et al., 2016)
	Wheat	Bran	Spray drying at 130◦C	↑257.69 %F	-	-	-	-	-	-	(Belén Martín-Diana et al., 2021)
			Spray drying followed by addition pea protein isolate	↑130.77 %F	-	-	-	-	-	-	
	Rice	Bran	Freeze drying	-	-	↑71.96% T	-	-	-	-	(Laokuldilok and Kanha, 2017)
	Rice	Whole grain	150°C coated with	↓13.76%T		↓21.64% T	-	↓19.42%T	-	-	(Papillo et al., 2018)

Microencapsulation		rice	maltodextrins and gum Arabic (50:50, w/w)								
	Rice	Anthocyanins From whole grain rice	Cakes with 0.5%microencapsulated powders	↓50.25%T	↓66.42%T	↓33.33% T	-	↑4.32%T	-	-	(Aprodu et al., 2019)
	Rice	Bran	6.01% of starch concentration at 168.78°C and 4.96 MPA nozzle pressure	-	-	↑24.55% T	-	↑6.76%T	-	-	(Das et al., 2019)
	Rice	Bran	Spray drying at 140, 160 and 180°C	-	-	-	-	↑34.34%T (140°C); ↑47.95%T (160°C); ↑35.85%T (180°C);	-	-	Laokuldilok and Kanha, 2017)
	Rice	Bran	Freeze drying	-	-	-	-	↑13.94%	-	-	
	Rice	Whole grain	Aqueous or ethanolic extract followed by freeze-drying	↑517.74%T(AE); ↓55.73%T(EE)	↑161.79%T(AE); ↓90.72%T(EE)	↓79.43% T(AE); ↓41.16% T(EE)	-	-	↑8.94%T (AE); ↓1.12%T (EE)	-	(Bolea et al., 2021)
Micronization	Wheat	Whole grain	Milled using a KMX-500 resulting in particle size <0.300 mm (micronized vs traditional flour)	↑434.41%T	-	↑26.96% T	-	-	-	-	(Martini et al., 2017)
			Dried micronized pasta vs traditional pasta	↑793.65%T		↑17.69% T	-	-	-	-	

			Cooked micronized pasta vs Traditional pasta	↑866.36%T	-	↑94.65% T	-	-	-	-	
	Buckwheat	Hull	Milled through 40-mesh screen	↑42.70%T			↑23.33 %T	↑2.86%T	↑12.42%T	-	(Zhu et al., 2014)
Microfluidisation	Wheat	Bran	3 passes through 200 µm (IC200) and 5 passes through 87 µm (IC87)	↓52.94%F; ↑62.03%B; ↑31.85%T	-	-	↑185.07%T	↑304%T	↑280%T	-	(Wang et al., 2013)
	Corn	Bran	5 passes through 87 µm (IC87)	↓26.57%F; ↑44.28%B; ↑31.99%T	-	-	↑157.73%T	↑199.12%T	↑175.00%T	-	(Wang et al., 2014)
	Corn	Bran	5 passes with 158.7MPA and 145.9MPA	↑48.80 %F	-	-	-	↑30.50%T	↑34.21%T	-	(He et al., 2016)
Nixtamalization	Sorghum	Whole grain	1.13% of lime and 31 mins cooking	↓33.87%T (White sorghum); ↓74.747%T (red sorghum)	↓75%T (White sorghum); ↓50%T (red sorghum)	-	-	↓26.9%	↓49.9%	-	(Gaytán-Martínez et al., 2017)
			1% (w/w) of calcium carbonate salt	↑14.29%T; ↑2.55%F; ↑43.90%B	-	-	-	-	-	-	
Nixtamalization and extrusion	Corn Sorghum	Whole grain (corn), bran (Sorghum)	0.3% lime followed by extrusion at feed rate 45 RPM and screw speed 112 RPM	-	-	-	-	↓18.16%T (ENCF); ↓62.36% T (Tortillas, ENCF); ↑8.32%T (ENCF with sorghum added after extrusion); ↑49.23%T (Tortillas, ENCF with sorghum added after extrusion)	-	-	(Buitimea-Cantúa et al., 2018)

Abbreviation: Values are %change from first to last data point, lowest to highest concentration, or control versus variables: 2,2'-azino-bis (3-ethylbenzothiazoline-6-sulfonic acid) (ABTS); Aqueous Extract (AE); 2,2-diphenyl-1- picryl-hydrazyl-hydrate (DPPH); Bound (B); Black Colored Rice (BCR); Ethanolic Extracts (EE); Extruded Nixtamalized Corn Flour (ENCF); F (Free); Ferric reducing antioxidant power (FRAP); *L. lactis ssp. Lactis* (UGAL2); *W. confusa* (UGAL1); *L. plantarum* (15GAL); *L. brevis* (16GAL); *L. plantarum*; *L. brevis* (DI-PROX MTTX), *L. helveticus* (LH-B02); *K. Marxianus* subsp.Marxianus

(LAF-4); ND (Not Detected); RCR (Red Colored Rice); Relative Humidity (RH); RPM (Revolutions Per Minute); SC (Soluble Conjugate); T (Total); Total Anthocyanin Content (TAC); Total Flavonoid Content (TFC); Total Phenolic Content (TPC); Ultrasonic Intensity (UI); White Rice (WR).
